# Galantamine ameliorates experimental pancreatitis

**DOI:** 10.1186/s10020-023-00746-y

**Published:** 2023-10-31

**Authors:** Dane A. Thompson, Tea Tsaava, Arvind Rishi, Sam J. George, Tyler D. Hepler, Daniel Hide, Valentin A. Pavlov, Michael Brines, Sangeeta S. Chavan, Kevin J. Tracey

**Affiliations:** 1 Laboratory of Biomedical Sciences, Institute for Bioelectronic Medicine, Feinstein Institutes for Medical Research, Northwell Health, 350 Community Drive, Manhasset, NY 11030 USA; 2 The Elmezzi Graduate School of Molecular Medicine, Manhasset, NY USA; 3 Donald and Barbara Zucker School of Medicine at Hofstra/Northwell, Hofstra University, Hempstead, NY USA; 4 Department of Surgery, Northshore University Hospital, Northwell Health, Feinstein Institutes for Medical Research, Manhasset, NY USA; 5 Department of Pathology and Laboratory Medicine, Donald and Barbara Zucker School of Medicine at Hofstra/Northwell, Hempstead, NY USA

**Keywords:** Pancreatitis, Galantamine, Vagus nerve, Inflammation, Cytokines, Cholinergic anti-inflammatory pathway

## Abstract

**Background:**

Acute pancreatitis is a common and serious inflammatory condition currently lacking disease modifying therapy. The cholinergic anti-inflammatory pathway (CAP) is a potent protective anti-inflammatory response activated by vagus nerve-dependent α7 nicotinic acetylcholine receptor (α7nAChR) signaling using splenic CD4^+^ T cells as an intermediate. Activating the CAP ameliorates experimental acute pancreatitis. Galantamine is an acetylcholinesterase inhibitor (AChEI) which amplifies the CAP via modulation of central muscarinic ACh receptors (mAChRs). However, as mAChRs also activate pancreatitis, it is currently unknown whether galantamine would be beneficial in acute pancreatitis.

**Methods:**

The effect of galantamine (1–6 mg/kg-body weight) on caerulein-induced acute pancreatitis was evaluated in mice. Two hours following 6 hourly doses of caerulein (50 µg/kg-body weight), organ and serum analyses were performed with accompanying pancreatic histology. Experiments utilizing vagotomy, gene knock out (KO) technology and the use of nAChR antagonists were also performed.

**Results:**

Galantamine attenuated pancreatic histologic injury which was mirrored by a reduction in serum amylase and pancreatic inflammatory cytokines and an increase the anti-inflammatory cytokine IL-10 in the serum. These beneficial effects were not altered by bilateral subdiaphragmatic vagotomy, KO of either choline acetyltransferase^+^ T cells or α7nAChR, or administration of the nAChR ganglionic blocker mecamylamine or the more selective α7nAChR antagonist methyllycaconitine.

**Conclusion:**

Galantamine improves acute pancreatitis via a mechanism which does not involve previously established physiological and molecular components of the CAP. As galantamine is an approved drug in widespread clinical use with an excellent safety record, our findings are of interest for further evaluating the potential benefits of this drug in patients with acute pancreatitis.

**Supplementary Information:**

The online version contains supplementary material available at 10.1186/s10020-023-00746-y.

## Introduction

Acute pancreatitis is a prevalent medical problem accounting for more than 275,000 hospital admissions each year in the United States and frequently complicated by serious morbidity and mortality (Li et al. 2021). Pancreatic damage is initiated by the abnormal release of digestive enzymes into the pancreatic parenchyma by acinar cells (Gukovskaya et al. 2017). At sites of tissue injury, damage-associated molecular patterns (DAMPs, e.g. high mobility group box-1 (HMGB1)) (Kang et al. 2014) and pro-inflammatory cytokines and chemokines are synthesized and released (Greer et al. 2022). These molecules recruit neutrophils and macrophages into the pancreas, perpetuating the inflammatory response in a feed-forward loop (Jakkampudi et al. 2017). Current treatment strategies for acute pancreatitis focus on supportive care (Lankisch et al. 2015), as no approved therapy addresses the underlying pancreatic inflammatory process, with the possible exception of rectal indomethacin administration prior to endoscopic retrograde cholangiopancreatography (ERCP) for select patients (Levenick, et al. 2016; Abdelfatah et al. 2020).

As inflammation is inherently a self-amplifying process, endogenous mechanisms exist which are typically engaged to limit collateral damage to normal tissues. For example, the inflammatory reflex is an important protective response activated when vagus nerve sensory fibers detect the presence of pro-inflammatory molecules. Afferent vagus nerve fibers then signal to the brain via the nucleus tractus solitarius (Pavlov et al. 2003). A response is propagated back to the periphery via cholinergic vagus efferent neurons, which subsequently activate intermediary, choline acetyltransferase (ChAT)-positive T-helper lymphocytes within the spleen (Rosas-Ballina et al. 2011). These T-cells release acetylcholine (ACh), activating α7 nicotinic acetylcholine receptors (α7nAChR) of macrophages (Wang et al. 2003), inhibiting production and release of pro-inflammatory cytokines. This efferent arc of the inflammatory reflex is referred to as the cholinergic anti-inflammatory pathway (CAP) (Tracey 2009).

The vagus nerve is known from prior work to play a protective role in the setting of acute pancreatitis. For example, a previous study has shown that pancreatitis worsens following vagotomy or use of a nAChR antagonist, while in contrast the α7nAChR agonist GTS-21 protects via inhibition of macrophage pro-inflammatory cytokine production (Westerloo et al. 2006). Additionally, the results of a recent study demonstrated that direct optogenetic stimulation of the efferent vagus nerve at the level of the dorsal motor nucleus (DMN) in the brainstem significantly reduces the severity of acute pancreatitis via a nAChR-dependent mechanism (Thompson et al. 2023). These observations demonstrate the potential therapeutic relevance of activating neuronal cholinergic pathways to reduce the severity of acute pancreatitis. However, a lack of FDA approval for GTS-21 and the invasive nature of DMN stimulation using current technology present significant challenges for clinical translation.

There are, however, approved cholinergic agents in clinical use. For example, galantamine is currently used to treat Alzheimer’s disease (Thompson 2001). This alkaloid derived from a member of the Amaryllis family is a potent, reversible acetylcholinesterase inhibitor (AChEI) (Sramek et al. 2000) which also sensitizes nAChRs to ACh (Wang et al. 2007). Prior studies have shown that the CAP is activated by galantamine via increased muscarinic ACh receptor (mAChR)-dependent brain signaling via its AChEI activity (Ji et al. 2014; Pavlov et al. 2009). To date, galantamine has demonstrated protective effects across diverse preclinical disease models of inflammatory diseases (Metz and Pavlov 2021) and has shown efficacy in a clinical trial of patients with metabolic syndrome, reducing markers of oxidative stress and systemic inflammation (Consolim-Colombo 2017; Sangaleti et al. 2021).

However, despite studies demonstrating the protective effects of the CAP in models of acute pancreatitis, the role of acetylcholine esterase inhibitors in the context of pancreatitis is still controversial (Ikeda et al. 2013; Yang et al. 2022). The potential benefit of repurposing a drug already in widespread clinical use with a low rate of associated side effects prompted our evaluation of galantamine utilizing caerulein-induced acute pancreatitis in mice. The results show that galantamine provides significant anti-inflammatory and pancreas-protective benefits in this setting, but by a mechanism not dependent upon the CAP or nAChR signaling.

## Methods and materials

### Animals and experimental model

All procedures and experiments were approved, in accordance with NIH guidelines, by the Institutional Animal Care and Use Committee and the Institutional Biosafety Committee of the Feinstein Institutes for Medical Research, Northwell Health, Manhasset, NY. Animals (8–12 week old, male and female) were maintained at 25 °C on a 12-h light–dark cycle, and allowed free access to food and water. Mice that underwent vagotomy were fasted for 3 h prior to surgery. C57BL/6 J (strain #:000664) mice were purchased from Jackson Lab (Bar Harbor, ME). CD4-cre (Tg(Cd4-cre)1Cwi/BfluJ, strain #: 017336) and ChAT^fl/fl^ (B6;129-Chat^tm1Jrs/J^, strain #: 16920) were purchased from Jackson Lab and crossed; CD4-cre x ChAT^fl/fl^ (CD4/ChAT^fl/fl^) were used for experiments along with ChAT^fl/fl^ littermate controls. Alpha-7 nicotinic acetylcholine receptor (α7nAChR) knockout (KO) (B6.129S7-Chrna7^tm1Bay^/J, stain #: 003232) were purchased and maintained in house; C57BL/6 J (strain # 000664) mice were used in place of littermate controls. Acute pancreatitis was induced by 6 hourly intraperitoneal injections (i.p.) of caerulein (Sigma, C9026). A dose of 50 µg/kg-body weight (bw) was used for all injections. Control mice received 6 hourly i.p. 0.9% saline injections. Mice were euthanized 2 h after the last injection.

### Drug treatment

Galantamine hydrobromide (Calbiochem, # 345670) was administered at 1, 4, or 6 mg/kg-bw i.p. at 1 h before the first dose of caerulein and again 30 min after the third dose. This provides approximate human equivalent doses calculated allometrically (Nair and Jacob 2016) to be 0.08, 0.33, and 0.49 mg/kg-bw respectively, which spans the normal dosing range of humans receiving treatment for Alzheimer’s disease (0.13 to 0.40 mg/kg-bw). Vehicle-injected control mice received identical volumes of sterile 0.9% saline. Mecamylamine hydrochloride (Sigma-Aldrich, M9020) or methyllycaconitine citrate (Sigma-Aldrich, M168) was administered i.p at a dose of 1 mg/kg-bw 2 h prior to the induction of acute pancreatitis, 1 h prior to administration of galantamine. A second dose was administered 4 h later.

### Subdiaphragmatic vagotomy and pyloric dilation

Mice were anesthetized with isoflurane (2% induction, 1.5% maintenance). Midline celiotomy was performed and small bowel was placed in the right lower quadrant. A small 4–5 mm incision was made on the greater curvature of the stomach in an area without obvious perforating blood vessels. Vascular dilators were coated in water-based lubricant, then introduced through the gastrotomy and passed through the pyloric sphincter. This was repeated 6 times with successively larger dilators, 1.5–4 mm, increasing 0.5 mm with each dilation. Gastrotomy was closed in a running fashion with absorbable suture (Vicryl 6.0). The stomach was then retracted inferiorly exposing the esophagus. Anterior and posterior branches of the vagus nerve were isolated and ligated just below the diaphragmatic hiatus. The celiotomy was closed in layers with absorbable suture (Vicryl 5.0) and surgical staples. Sham surgery mice underwent an identical pyloric dilation, but the vagus nerve was not manipulated. All mice received 1 ml of warm, sterile 0.9% saline subcutaneously prior to being placed in a clean recovery cage. The mice recovered for at least 7 days prior to additional experimentation.

### Tissue handling, histologic examination and assays

Following euthanasia, blood was collected via cardiac puncture and serum isolated by centrifugation. Pancreas tissue for enzyme-linked immunosorbent assay (ELISA) was immediately placed in tissue protein extraction reagent (Thermo Scientific, 78510) with protease inhibitor (Thermo Scientific, A32953) and homogenized. Serum and tissue were stored at – 80 °C. Pancreas for histological examination was fixed in neutral buffered formalin (Sigma, HT5011). Paraffin-embedded sections of pancreas were stained with hematoxylin and eosin (H&E). Samples were evaluated for edema, acinar necrosis, hemorrhage and fat necrosis, and inflammation and perivascular infiltration. Severity of acute pancreatitis was graded according to previously established criteria by a pathologist blinded to groups (Schmidt et al. 1992). A sum of these individual scores was reported as a total score. Serum amylase activity was measured via colorimetric enzymatic assay (Abcam, ab102523). Serum and pancreatic cytokine concentrations were measured by ELISA (Meso Scale Diagnostics, V-Plex).

## Statistical analysis

All statistical analyses were performed using GraphPad Prism (GraphPad Software, v 9.3.0). Normality was determined with Shapiro–Wilk testing. For parametric data sets, student’s t-testing or one-way ANOVA testing was used. For non-parametric data sets, Mann–Whitney U-testing or Kruskal–Wallis testing was used. Statistical significance was defined as p ≤ 0.05 for a two tailed distribution.

## Results

### Administration of galantamine reduces the severity of pancreatitis

**Fig. 1:**
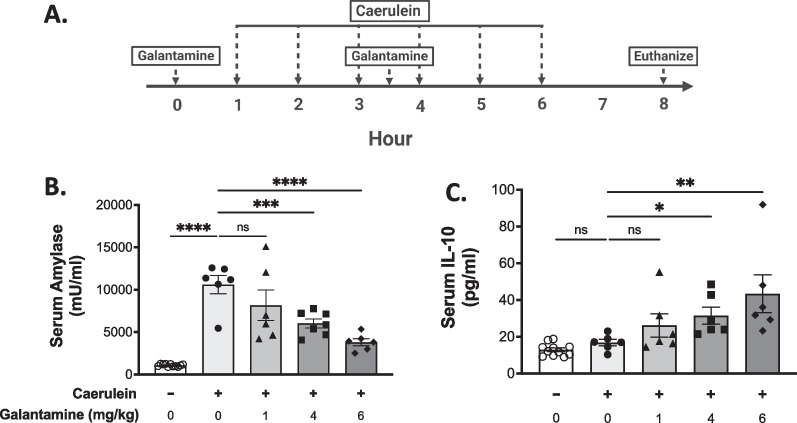
Galantamine improves serum markers of acute pancreatitis severity. **A** Acute pancreatitis was induced by 6 hourly intraperitoneal injections of 50 µg/kg-bw caerulein. Galantamine (1, 4, or 6 mg/kg-bw), or vehicle, was administered one hour prior to the first injection of caerulein and again after the third injection. Mice were euthanized two hours after the final injection of caerulein. Serum collected at the time of euthanasia demonstrates a dose-dependent **B** decrease in serum amylase and **C **increase in IL-10. Data are presented as individual mouse data points with mean ± SEM. One-Way ANOVA with Kruskal–Wallis, *p < 0.05, **p < 0.01, ***p < 0.001, ****p ≤ 0.0001, ns = not significant. n = 6–11

Here, we studied whether galantamine improves disease severity in caerulein-induced pancreatitis. Administration of galantamine significantly attenuated serum amylase, one marker of pancreatic inflammation, while increasing the tissue reparative cytokine IL-10 at 4 and 6 mg/kg doses (Fig. 1A–C). In pancreatic tissue, levels of the pro-inflammatory chemokine MCP-1 and cytokines IL-β were significantly reduced, as well as a strong trend for reduced TNF, and increased IL-10 (Additional file 1: Fig. S1).

**Fig. 2:**
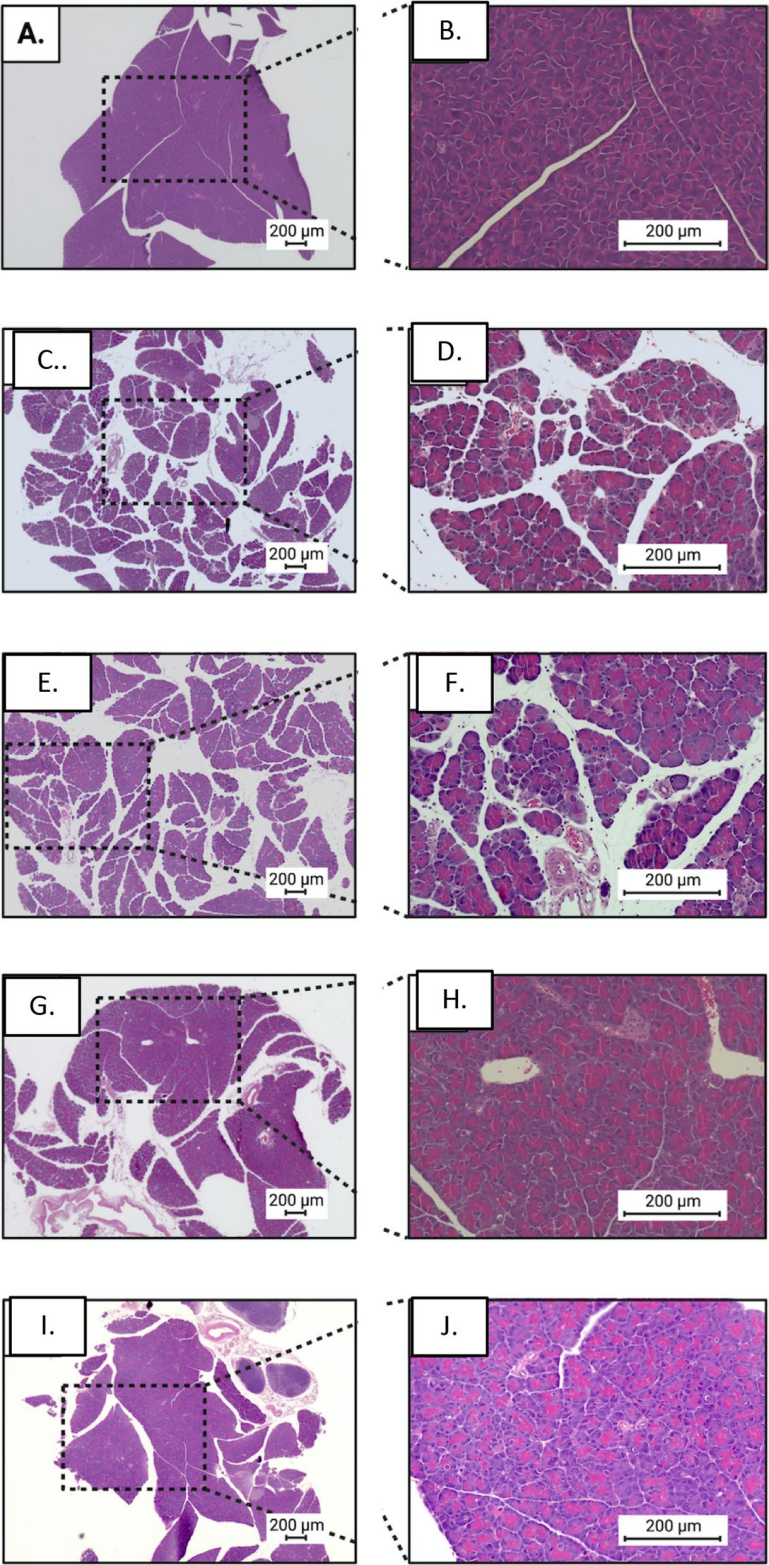
Administration of Galantamine mitigates the histological severity of acute pancreatitis. **A**–**J** Representative images of pancreatic tissue stained with H&E at 4x (left panels) and 20x (right panels). **A**, **B** Mice without induction of pancreatitis, **C**, **D** pancreatitis and vehicle, **E**, **F** pancreatitis and galantamine (1 mg/kg-bw), **G**, **H** pancreatitis and galantamine (4 mg/kg-bw), and **I**, **J** pancreatitis and galantamine (6 mg/kg-bw). Bar = 200 μm

**Fig. 3:**
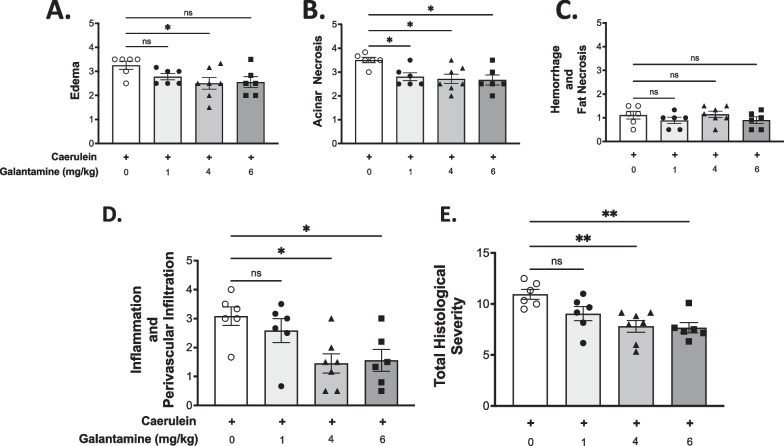
Administration of galantamine reduces the histological severity of acute pancreatitis. Histological scoring for (**A**) edema, (**B**) acinar necrosis, (**C**) hemorrhage and fat necrosis, (**D**) inflammation and perivascular infiltration, and (**E**) total severity. Data are represented as individual mouse data points with mean ± SEM. One-Way ANOVA with Kruskal–Wallis, *p < 0.05, **p < 0.01, ns = not significant. n = 5–6

That the severity of pancreatitis is decreased following galantamine administration was further demonstrated by significant reduction in the histological manifestations of the disease (Fig. 2). The degree of tissue edema was significantly reduced in the 4 mg/kg-bw galantamine group (Fig. 3A), a dose which has been previously shown to inhibit mouse AChE activity by 43% (Bickel et al. 1991), and suppress inflammation in preclinical models (Ji et al. 2014; Pavlov et al. 2009). A significant reduction in the acinar necrosis was observed for all doses of galantamine (Fig. 3B). A mild degree of hemorrhagic and fat necrosis, typical for caerulein-induced pancreatitis, was observed in all the experimental groups (Fig. 3C). Galantamine administration also significantly attenuated inflammation and perivascular infiltration at 4 mg/kg-bw and 6 mg/kg-bw doses (Fig. 3D). Accordingly, the total histology severity, a sum of scores from the previous four categories, was significantly lower in the 4 mg/kg-bw and 6 mg/kg-bw groups (Fig. 3E).

### Galantamine reduces pancreatitis severity in a vagus nerve-independent manner

**Fig. 4:**
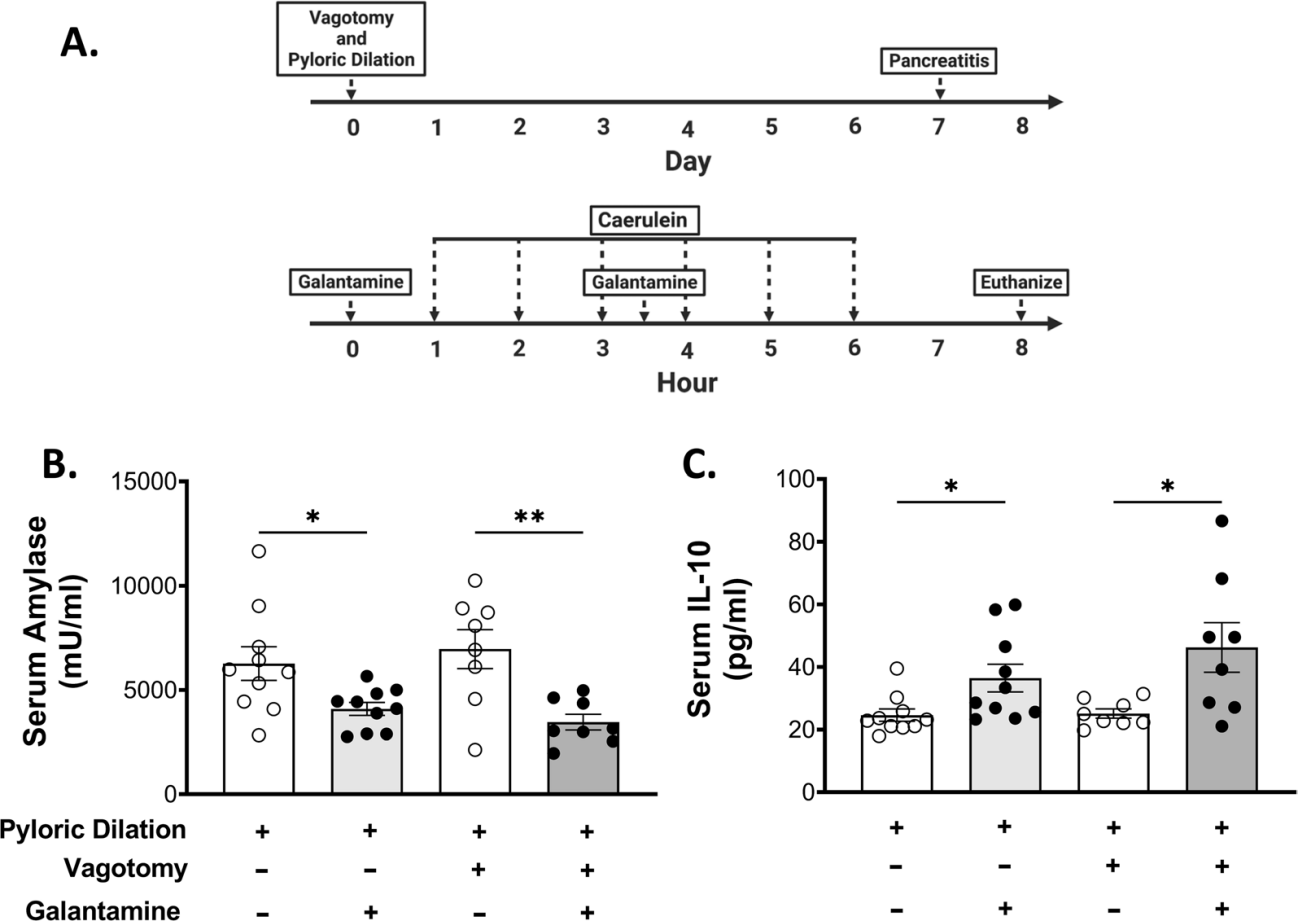
Galantamine decreases pancreatitis severity in a vagus nerve-independent manner. **A** Mice underwent bilateral, subdiaphragmatic vagotomy, or sham operation, then were allowed to recover for 1 week prior to the induction of pancreatitis. Galantamine administration significantly reduces serum amylase (**B**) and elevates serum IL-10 (**C**) in sham and vagotomy animals. Data are represented as individual mouse data points with mean ± SEM. Unpaired t-test, *p < 0.05, **p < 0.01, ns = not significant. n = 8–10

Previous studies have demonstrated that galantamine is a centrally-acting acetylcholinesterase inhibitor that significantly increases brain cholinergic network activity (Reichman 2003; Ellis 2005), resulting in activation of vagus nerve outflow to trigger the CAP (Waldburger et al. 2008). To assess whether the beneficial effects of galantamine in the context of pancreatitis depend on an intact vagus nerve, we studied mice subjected to vagus nerve transection. Animals were subjected to bilateral subdiaphragmatic vagotomy (Fig. 4A), eliminating all vagus nerve signaling to the abdominal organs. Vagotomy did not alter the development of pancreatitis and did not abolish the protective effects of galantamine, as similar to sham surgery controls, galantamine (4 mg/kg-bw) reduced serum amylase levels and increased serum IL-10 (Fig. 4B, C). This observation shows that vagus nerve signaling is not required for the beneficial effects of galantamine in caerulein-induced pancreatitis.

### Acetylcholine-producing T cells are not required for the protective effects of galantamine in pancreatitis

**Fig. 5:**
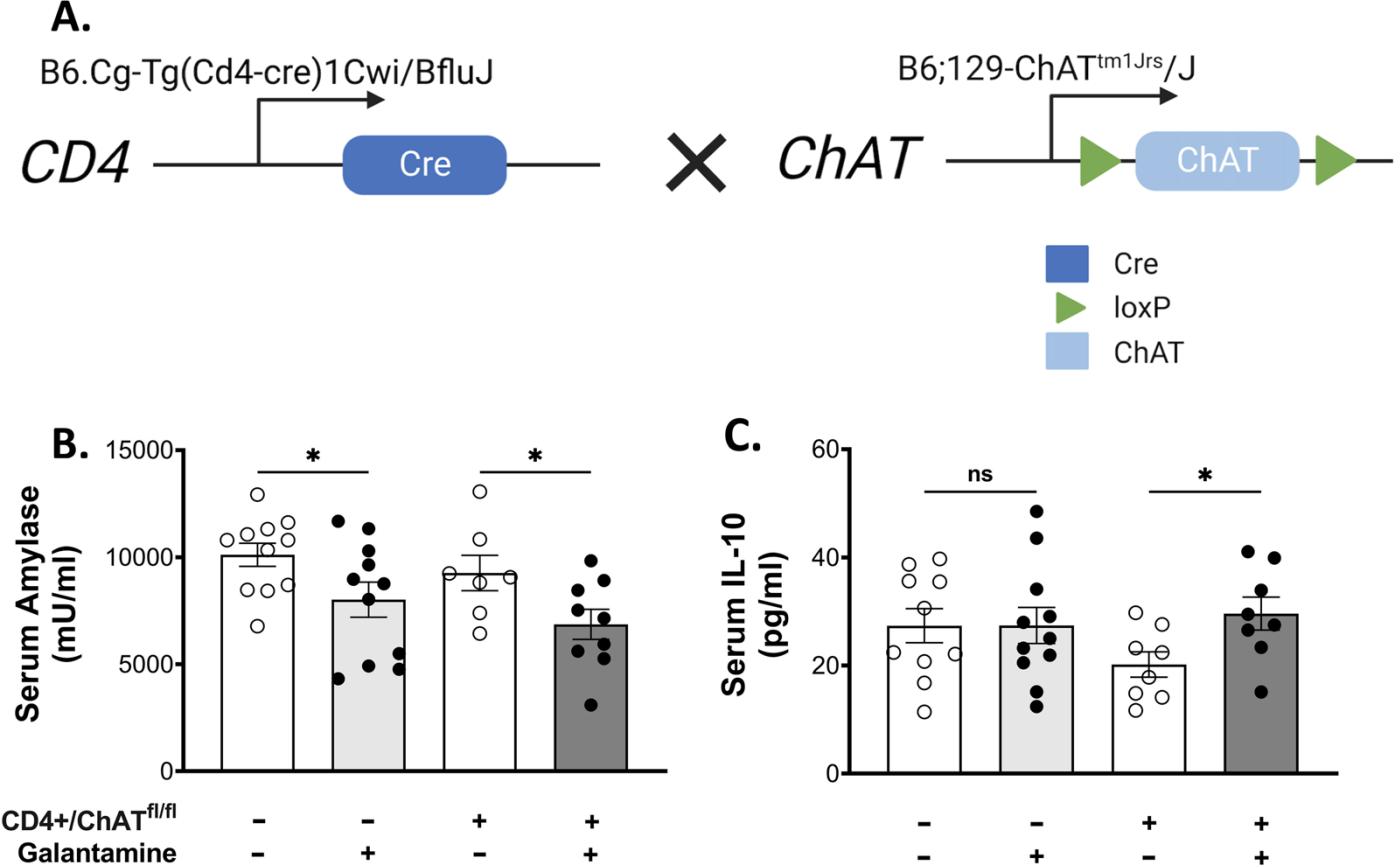
Choline acetyltransferase expressing T-cells are not required for galantamine-mediated effects in acute pancreatitis. **A** Breeding strategy for CD4^+^/ChAT^fl/fl^ mice. Galantamine administration significantly reduces serum amylase (**B**) and elevates serum IL-10 (**C**) in CD4^+^/ChAT^fl/fl^ and littermate controls. Data are represented as individual mouse data points with mean ± SEM. Unpaired t-test, *p < 0.05, ns = not significant. n = 10–11

The protective effects of galantamine are functionally associated with signaling via the splenic nerve and result in an increase of ACh within the spleen (Ji et al. 2014; Pavlov et al. 2009). To determine whether ACh-producing T-cells are required for mediating the protective effects of galantamine in acute pancreatitis, we selectively ablated ChAT expression in T-cells by crossing CD4-Cre mice with floxed *ChAT* mice (ChAT^fl/fl^) to generate offspring deficient in ChAT-expressing-T cells (CD4^+^/ChAT^fl/fl^) (Fig. 5A). Littermate controls (ChAT^fl/fl^) and age-matched CD4^+^/ChAT^fl/fl^ mice were injected i.p. with galantamine (4 mg/kg-bw) or vehicle (saline) 1 h before the first dose of caerulein and 30 min after the third dose. Galantamine significantly suppressed serum amylase levels and increased serum IL-10 levels in both WT and CD4^+^/ChAT^fl/fl^ mice receiving cerulein (Fig. 5B, C), indicating that ChAT expressing T-cells are not required for the protective effects of galantamine in pancreatitis.

### Nicotinic acetylcholine receptors do not mediate the protective effects of galantamine in pancreatitis

**Fig. 6:**
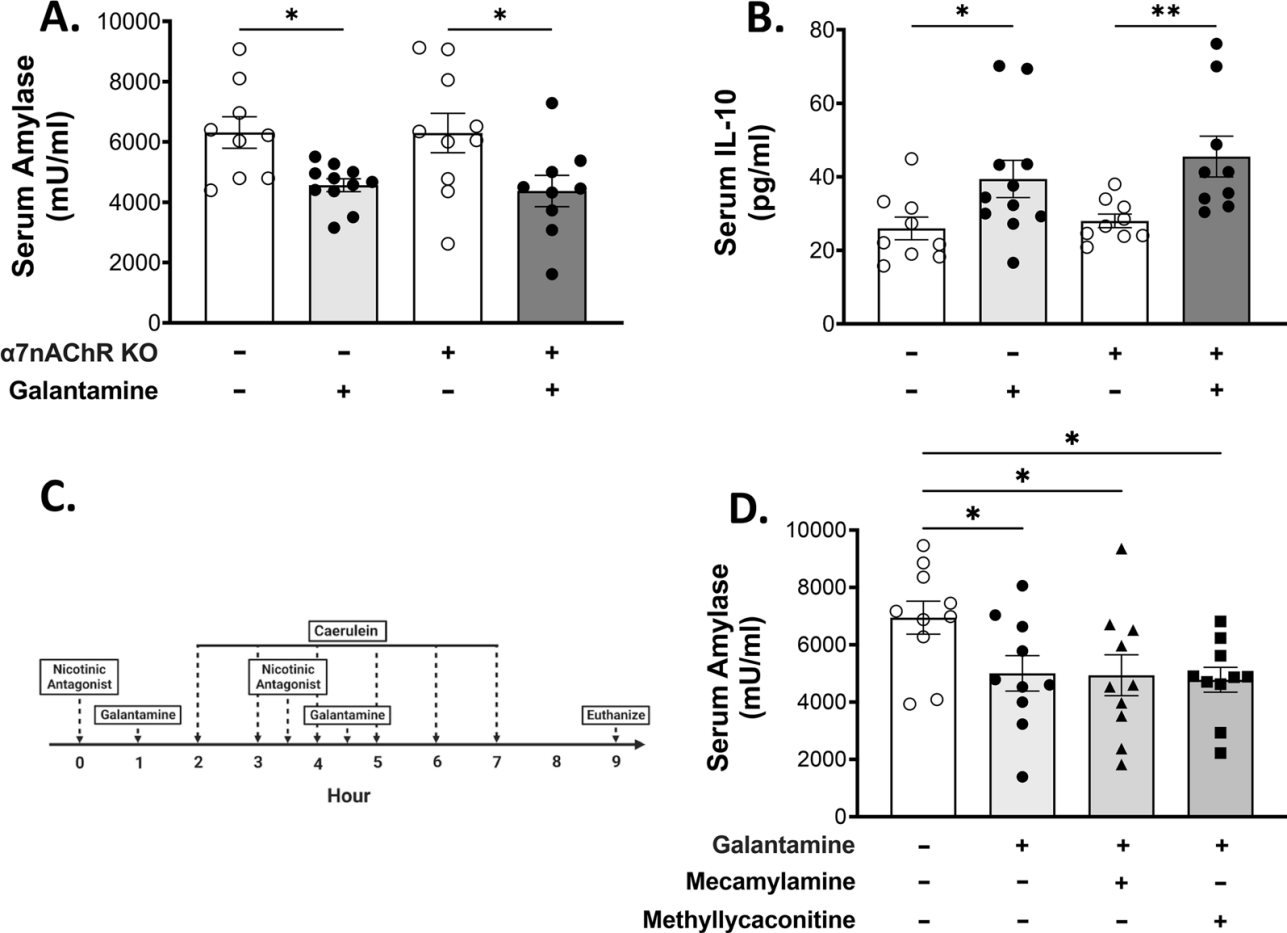
Nicotinic acetylcholine receptors are not required for galantamine-mediated amelioration of acute pancreatitis. Galantamine administration significantly reduces serum amylase (**A**) and elevates serum IL-10 (**B**) in α7nAChR KO and WT mice. **C**, **D** Pre-treatment with nicotinic antagonists, mecamylamine (1 mg/kg-bw) or methyllycaconitine (1 mg/kg-bw) does not alter galantamine-mediated suppression of serum amylase. Data are represented as individual mouse data points with mean ± SEM. Unpaired t-test, *p < 0.05, **p < 0.01, ns = not significant. n = 10–11

Galantamine is a positive allosteric modulator of nicotinic acetylcholine receptors, including α7nAChR (Wang et al. 2007), which is required for reduced cytokine production via the CAP (Tracey 2009). Therefore, we evaluated the efficacy of galantamine in attenuating disease severity in caerulein-induced pancreatitis in WT and α7nAChR KO mice. Galantamine administered to α7nAChR KO mice resulted in significantly decreased serum amylase levels and increased serum IL-10 levels, similar to WT controls (Fig. 6A, B).

Having established that genetic ablation of α7nAChR does not alter the protective effects of galantamine in pancreatitis, we next determined whether pharmacologic silencing of nicotinic receptors influences pancreatitis severity. Separate cohorts of WT mice were subjected to 6 hourly injections of caerulein to induce pancreatitis after i.p. injection of the ganglionic blockers mecamylamine (a nicotinic receptor antagonist) and methyllycaconitine (a predominantly α7nAChR antagonist) compared to vehicle (Fig. 6C). In line with the results obtained with α7nAChR KO mice, blocking of  nicotinic receptors using mecamylamine or selective blocking of α7nAChR with methyllycaconitine did not alter the protective effects of galantamine (Fig. 6D). Together these results of these experiments demonstrate that galantamine mediates its protective effects in caerulein-induced pancreatitis independent of vagus nerve signaling, ChAT^+^ T cells and nAChRs.

## Discussion

The results of this study show that galantamine ameliorates acute pancreatitis via activation of an anti-inflammatory program and does not amplify injury via an anticholinesterase-mediated increase in local ACh and mAChR processes within the pancreas (Yang et al. 2022; Wan 2021). The observed attenuation of serum amylase coupled with an increase in the anti-inflammatory cytokine IL-10 and reduced pancreatic inflammatory mediators are biologically significant effects as shown by the improved histological markers of pancreatic injury. Importantly, this beneficial action of galantamine occurs at equivalent human doses within the range employed by current clinical practice for treatment of Alzheimer’s disease. This potentially minimizes the possibility of significant adverse effects, as the decades-long clinical experience using galantamine has shown it to be an exceptionally safe drug (Metz and Pavlov 2021). Therefore, our observations support evaluating galantamine for the treatment of early, developing acute pancreatitis, as well as potentially to be administered prophylactically in clinical settings in which there is risk of procedure-related pancreatic injury, e.g., following endoscopic retrograde cholangiopancreatography (ERCP) which occurs in up to 40% of high risk patients (Thaker et al. 2015).

A limitation of this study is that the mechanism of the beneficial effects of galantamine on pancreatitis is currently unresolved. Our observations that subdiaphragmatic vagotomy, which severs vagus nerve innervation of the pancreas does not abrogate galantamine effects and the persistence of drug’s effects in mice lacking ChAT^+^ T cells or α7nAChR  indicate that galantamine is mediating its protective effects independent of CAP. Additionally, as both the parasympathetic and sympathetic parts of the autonomic nervous system use ACh as a neurotransmitter at the level of the ganglia, the lack of effects of two different nicotinic blockers, one non-selective (mecamylamine) and one more selective for α7nAChR (methyllylaconitine), effectively rule out contributions from the autonomic nervous system. This includes sympathetic outflow which has been shown to provide robust anti-inflammatory activity, as post ganglionic transmission within both branches of the autonomic nervous system is blocked by these compounds (Biaggioni 2017; Kaushal and Tadi 2023). Of note, the theoretical possibility of not achieving a full ganglionic transmission blockade using these doses of antagonists should be considered. Although galantamine has reported effects on other neurotransmitters, e.g., dopamine and serotonin, which may have beneficial actions on pancreatic function and pancreatitis (Han et al. 2017; Owyang and Logsdon 2004), these effects may be secondary to potentiating effects on nAChR function, particularly presynaptically via the α7nAChR, a possibility which is ruled out by the nAChR antagonists employed and the gene KO experiments. Additionally, galantamine has anti-oxidant effects (Tsvetkova et al. 2013) which could affect the development acute pancreatitis which depends in part upon free radical-induced tissue injury. However, these antioxidant effects may depend on cholinergic sensitizing effects (Tsvetkova et al. 2013) and, further, the dosages of galantamine required for this effect are likely to be higher than those used in the current study.

One potential mechanism that may play a role in mediating the anti-inflammatory effects of galantamine in the setting of ganglionic blockade could be theoretically linked to a humoral pathway. In this scenario, inflammatory mediators released during caerulein-induced pancreatitis travel within the blood to the carotid bodies which act as immunosensors (Katayama et al. 2022). Afferent signaling then travels centrally via the carotid sinus nerve into the paraventricular nucleus (PVN) (Falvey et al. 2020). Activation of corticotropin releasing factor (CRF) producing neurons in the dorsal part of the PVN depends in part on muscarinic receptors (Mukai et al. 2020), which galantamine enhances via increased availability of ACh (Pavlov et al. 2009). CRF released into the median eminence then enhances release of glucocorticoids in circulation through the hypothalamic–pituitary–adrenal axis. Glucocorticoid receptor activation on myeloid cells would protect from pancreatitis via dampening the production of pro-inflammatory molecules (Falvey et al. 2020). Further study will be necessary to evaluate this possibility.

There are additional limitations of this study. Here, the effects of galantamine on the development of caerulein-induced pancreatitis were only evaluated at a single, early time point. The potential use of galantamine as treatment of more advanced acute pancreatitis warrants further study. Furthermore, approximately 20% of patients develop recurrent pancreatitis following an initial episode, and 35% of those patients will eventually develop chronic pancreatitis (Machicado and Yadav 2017). Therefore, study of galantamine in a model of repeated acute pancreatitis will be needed to evaluate its potential as a therapeutic to prevent the complications of chronic pancreatitis, such as exocrine insufficiency (Rijk et al. 2022) and diabetes (Woodmansey et al. 2017).

## Conclusion

In conclusion, our findings demonstrate that galantamine reduces the severity of acute pancreatitis at doses that were previously used in other inflammatory conditions and in preclinical models of Alzheimer’s disease. As a clinically approved drug with a large therapeutic index in widespread clinical use for decades, galantamine should be evaluated in clinical trials for potential disease modifying effects on acute pancreatitis.

### Supplementary Information

**Additional file 1: Figure S1.** Galantamine decreases pancreatic pro-inflammatory cytokine production in acute pancreatitis. Galantamine administration reduces pancreatic (A) MCP-1 and (D) IL-1β but not (B) IL-10 or (C) TNFα. Data are represented as individual mouse data points with mean ± SEM. Unpaired t-test, *p<0.05, ns = not significant. n = 5-6.

## Data Availability

All data supporting the findings of this study are available within the paper and its additional materials.
