# Correction: Galantamine ameliorates experimental pancreatitis

**DOI:** 10.1186/s10020-024-00814-x

**Published:** 2024-04-03

**Authors:** Dane A. Thompson, Tea Tsaava, Arvind Rishi, Sam J. George, Tyler D. Hepler, Daniel Hide, Valentin A. Pavlov, Michael Brines, Sangeeta S. Chavan, Kevin J. Tracey

**Affiliations:** 1 Laboratory of Biomedical Sciences, Institute for Bioelectronic Medicine, Feinstein Institutes for Medical Research, Northwell Health, 350 Community Drive, 11030 Manhasset, NY USA; 2 The Elmezzi Graduate School of Molecular Medicine, Manhasset, NY USA; 3 Donald and Barbara Zucker School of Medicine at Hofstra/Northwell, Hofstra University, Hempstead, NY USA; 4 Department of Surgery, Northshore University Hospital, Northwell Health, Feinstein Institutes for Medical Research, Manhasset, NY USA; 5 Department of Pathology and Laboratory Medicine, Donald and Barbara Zucker School of Medicine at Hofstra/Northwell, Hempstead, NY USA

Correction: Molecular Medicine (2023) 29:149 10.1186/s10020-023-00746-y.

Following publication of the original article (Thompson et al., 2023), we have been informed that author’s name Daniel Hide had a spelling.

The incorrect name is: Daniel Hyde.

The correct name is: Daniel Hide.

The original article has been corrected.
