# Supplementary material for: Galantamine ameliorates experimental pancreatitis
Source: Mol Med. 2023 Oct 31;29:149. doi: 10.1186/s10020-023-00746-y (PMC10617083; doi:10.1186/s10020-023-00746-y)
Supplement: Supplementary file 1 — Additional file 1: Figure S1. Galantamine decreases pancreatic pro-inflammatory cytokine production in acute pancreatitis. Galantamine administration reduces pancreatic (A) MCP-1 and (D) IL-1β but not (B) IL-10 or (C) TNFα. Data are represented as individual mouse data points with mean ± SEM. Unpaired t-test, *p<0.05, ns = not significant. n = 5-6. [file 10020_2023_746_MOESM1_ESM.docx]

**Figure S1. Galantamine decreases pancreatic pro-inflammatory cytokine production in acute pancreatitis.** Galantamine administration reduces pancreatic (A) MCP-1 and (D) IL-1β but not (B) IL-10 or (C) TNFα. Data are represented as individual mouse data points with mean ± SEM. Unpaired t-test, *p<0.05, ns = not significant. n = 5-6.

**
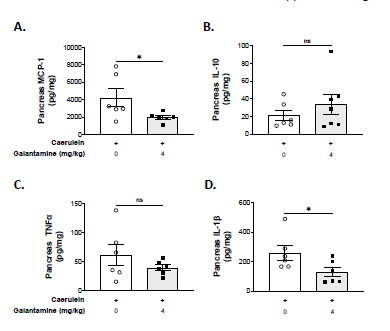
**
